# Efficacy of alendronate for the treatment of ankylosing spondylitis: A protocol for systematic review and meta-analysis

**DOI:** 10.1097/MD.0000000000021089

**Published:** 2020-07-24

**Authors:** Hua-yu Tang, Yu-zhi Li, Zhao-chen Tang, Quan-wei Jiang, Yu Zhao

**Affiliations:** a Second Ward of Orthopedics Department; b Department of Urology, First Affiliated Hospital of Jiamusi University; c School of Clinical Medicine, Jiamusi University, Jiamusi; d Department of Anesthesiology, Benxi Central Hospital of China Medical University, Benxi; e Department of Orthopedics, Huludao Central Hospital, Huludao, China.

**Keywords:** alendronate, ankylosing spondylitis, efficacy, safety

## Abstract

**Background:**

Ankylosing spondylitis (AS) is a very tricky orthopedic disorder. If such condition cannot be managed fairly well, it may significantly affect quality of life and even leads to disability among such population. A variety of studies have reported that alendronate is utilized for the treatment of AS. However, their results are still contrary, and no systematic review has addressed on this topic. Thus, this study will systematically assess the efficacy and safety of alendronate for the treatment of patients with AS.

**Methods:**

A comprehensive literature search will be performed from the below electronic databases from their commencement to the January 31, 2020 without language and publication status limitations: PubMed, Embase, Cochrane Library, Web of Science, Allied and Complementary Medicine Database, WANGFANG, and China National Knowledge Infrastructure. Only randomized controlled trials focusing on the alendronate for the treatment of patients with AS will be considered for inclusion in this study. Two authors will independently select all identified records, extract essential data from all included studies, and appraise study quality for each eligible trial using Cochrane risk of bias. If any differences occur, another experienced author will be invited to solve them by discussion and a consensus decision will be made. We will implement RevMan 5.3 software to analyze the extracted data.

**Results:**

This study will summarize high-quality randomized controlled trials to assess the efficacy and safety of alendronate for the treatment of patients with AS through primary outcome of bone densitometry; and secondary outcomes of pain intensity, quality of life, disease activity, functional status, and adverse events.

**Conclusion:**

This study will provide evidence to help determine whether alendronate is an effective and safe management for patient with AS or not.

**Study registration:**

INPLASY202040153.

## 1 Introduction

Ankylosing spondylitis (AS) is a chronic inflammatory rheumatic disease.^[1–3]^ It is characterized by inflammation of spine and the sacroiliac joints,^[4–6]^ which causes structural and functional impairments of those joints and results in poor quality of life.^[7–9]^ It is estimated that about 0.1% to 1.8% people affect this disorder and onset of symptoms commonly manifest in males between 15 and 25 years old.^[10,11]^ To date, its etiology remains not fully elucidated.^[12,13]^

Currently, very few medications can benefit patients with AS.^[14–16]^ Recent studies reported to use alendronate for the treatment of patients with AS.^[17–23]^ As far as we know, no previous systematic review has focused on the efficacy and safety of alendronate for treating AS. With the recent publication of high-quality studies on this subject,^[17–23]^ this study aims to systematically explore the efficacy and safety of alendronate for the treatment of patients with AS.

## 2 Methods and analysis

### 2.1 Study registration

We have registered this study on INPLASY202040153. It is developed according to the Preferred Reporting Items for Systematic Reviews and Meta-Analysis Protocol statement guidelines.^[24]^

### 2.2 Eligibility criteria for study selection

#### 2.2.1 Study types

This study will only include randomized controlled trials (RCTs) of alendronate for the treatment of patients with AS. Besides RCTs, we will exclude any other studies, such as nonclinical trials, non-RCTs, and quasi-RCTs.

#### 2.2.2 Intervention types

The experimental interventions are any forms of alendronate.

The control intervention could be any treatments, such as placebo, other medications, and alternative therapies. However, we will exclude studies using any types of alendronate, including its combination with other managements as their comparators.

#### 2.2.3 Patient types

Any adult participants (more than 18 years old) with a definite diagnosis of AS will be included in spite of their race, gender, educational background, and duration of AS.

#### 2.2.4 Outcome measurements

##### 2.2.4.1 Primary outcome

Bone densitometry (as reported in the trials by any instruments or tools).

##### 2.2.4.2 Secondary outcomes

Pain intensity (as reported in the trial by any pain scales)
Quality of life (as reported in the trial by any tools, such as Ankylosing Spondylitis Quality of Life questionnaire)
Disease activity (as reported in the trial by any indexes, such as Bath Ankylosing Spondylitis Disease Activity Index)
Functional status (as reported in the trial by any scores, such as Bath Ankylosing Spondylitis Functional Index)
Adverse events

### 2.3 Search strategy

#### 2.3.1 Electronic databases search

Two authors will carry out a comprehensive literature search from the below electronic databases: PubMed, Embase, Cochrane Library, Web of Science, Allied and Complementary Medicine Database, WANGFANG, and China National Knowledge Infrastructure. All these electronic databases will be searched from their commencement to the January 31, 2020 without language and publication status limitations. Any potential RCTs that explored the efficacy and safety of alendronate for the treatment of patients with AS will be included. An example of detailed search strategy for PubMed is presented (Table 1). We will modify similar search strategies for other electronic databases.

**Table 1:**
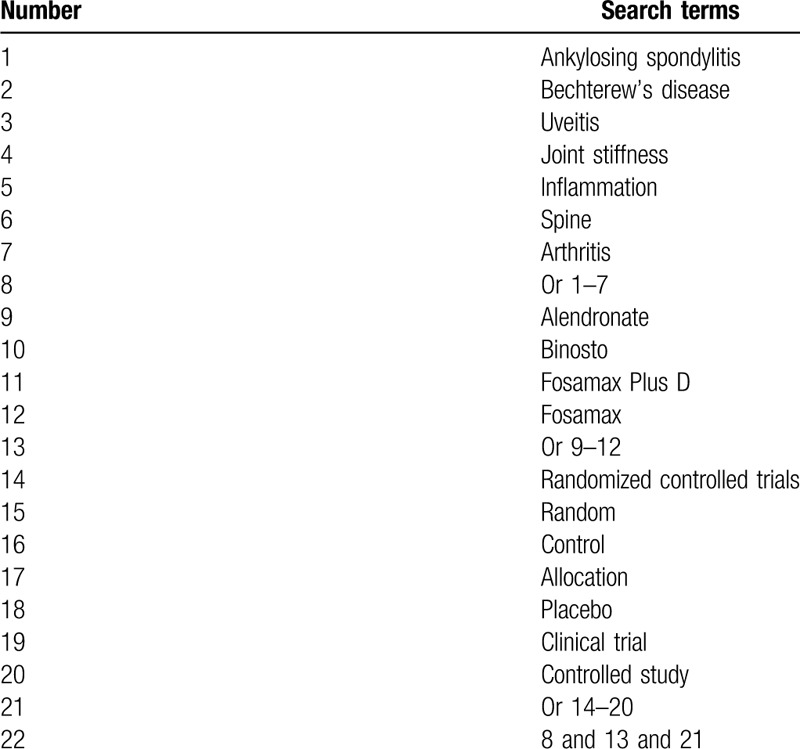
Search strategy of PubMed.

#### 2.3.2 Other literature sources search

We will search other literature resources, such as Google Scholar, websites of clinical trial registry, dissertations, and reference lists of included trials.

### 2.4 Literature selection

Records from all literature source searches will be imported into EndNote X9 to remove all duplicated studies. Two authors will firstly identify records by scanning titles and abstracts, and unrelated studies will be eliminated. Then, full-text of remaining studies will be read cautiously based on the predefined eligibility criteria. Any divergences will be figured out with the help of a third author. The process of study selection will be exerted in a flow diagram.

### 2.5 Data collection and management

Two authors will independently extract data from each included trial in accordance with the predefined data collection form. Any uncertainties will be resolved by another author and a consensus will be reached finally. The collected information is as follows:

Study details: title, first author, publication date, location, etc
Study methods: randomization specifics, blinding, allocation, follow-up information, etc
Patient characteristics: age, sex, sample size, duration and severity of AS, etc
Intervention and control details: treatment duration, dosage, frequency, etc
Outcomes: outcome measurements reported in the trial, adverse events, etc
Others: funding information, conflict of interest, etc

### 2.6 Dealing with unclear or missing data

Any unclear or insufficient data will be obtained from original authors with email or phone. If it is not achievable, we will perform data analysis based on the available data, and will discuss its impacts on study findings.

### 2.7 Risk of bias assessment

Study quality of each included trial will be evaluated by 2 independent authors using Cochrane risk of bias tool, which examines 7 domains. We will rate each source of bias as low, unclear, or high. Any different views will be worked out with the help of another author through discussion.

### 2.8 Treatment effect measurement

In this study, we will use mean difference or standardized mean difference and 95% confidence intervals (CIs) to evaluate the treatment effect for continuous data, and risk ratio and 95% CIs to appraise the treatment effect for dichotomous data.

### 2.9 Data analysis

RevMan 5.3 software will be employed for all data analysis and meta-analysis if it is possible. *I*^2^ test will be used for heterogeneity identification. *I*^2^ ≤ 50% suggests reasonable heterogeneity, and a fixed-effect model will be exerted. Otherwise, *I*^2^ > 50% means substantial heterogeneity, and a random-effects model will be employed. If ample data is extracted from sufficient trials with low heterogeneity, we will carry out meta-analysis to explain the results. On the contrary, we will perform subgroup analysis and sensitivity analysis to test possible sources of significant heterogeneity. If meta-analysis is deemed not to be conducted, we will elaborate study results using a narrative description.

#### 2.9.1 Subgroup analysis

Subgroup analysis will be developed to investigate the possible sources of significant heterogeneity according to the characteristics of study, types of intervention and controls, and different outcome measurements.

#### 2.9.2 Sensitivity analysis

A sensitivity analysis will be carried out to test the robustness and stability of pooled results by taking away low-quality trials or trials reporting data missing.

#### 2.9.3 Reporting bias

If it is possible to include at least 10 trials on one outcome, we will examine the possible reporting bias using funnel plot and Egger regression test.^[25,26]^

### 2.10 Dissemination and ethics

This study will be disseminated at a peer-reviewed journal or will be presented a scientific conference. This study will not collect original patient data, thus no ethic approval is needed.

## 3 Discussion

Alendronate is used to treat several osteoporosis diseases, including AS. For patients with AS, alendronate has been investigated in several clinical trials. However, its efficacy and safety on AS is still inconsistent. Hence, this study will assess the efficacy and safety of alendronate in patients with AS. This study may provide helpful evidence to judge whether alendronate is effective and safety in patients with AS.

This study still has several limitations. First, significant heterogeneity may exist, which may affect the accuracy of the findings. Second, AS severity and baseline characteristics of the patients may be vary among trials, this may impede its generation to all AS patients. Third, the number of eligible trials and patient samples may be relatively small, which may weaken the power of the present study.

## Author contributions

**Conceptualization:** Hua-yu Tang, Yu-zhi Li, Zhao-chen Tang, Quan-wei Jiang, Yu Zhao.

**Data curation:** Hua-yu Tang, Yu-zhi Li, Quan-wei Jiang, Yu Zhao.

**Formal analysis:** Hua-yu Tang, Zhao-chen Tang, Quan-wei Jiang, Yu Zhao.

**Investigation:** Yu Zhao.

**Methodology:** Hua-yu Tang, Yu-zhi Li, Zhao-chen Tang, Quan-wei Jiang.

**Project administration:** Yu Zhao.

**Resources:** Hua-yu Tang, Yu-zhi Li, Zhao-chen Tang, Quan-wei Jiang.

**Software:** Hua-yu Tang, Yu-zhi Li, Zhao-chen Tang, Quan-wei Jiang.

**Supervision:** Yu Zhao.

**Validation:** Hua-yu Tang, Yu-zhi Li, Quan-wei Jiang, Yu Zhao.

**Visualization:** Hua-yu Tang, Yu-zhi Li, Zhao-chen Tang, Quan-wei Jiang, Yu Zhao.

**Writing – original draft:** Hua-yu Tang, Yu-zhi Li, Zhao-chen Tang, Yu Zhao.

**Writing – review & editing:** Hua-yu Tang, Zhao-chen Tang, Quan-wei Jiang, Yu Zhao.
